# Rationale and design for the Blood Pressure Control Target in Diabetes (BPROAD) study

**DOI:** 10.1111/1753-0407.13411

**Published:** 2023-06-21

**Authors:** The BPROAD Study Group, Guang Ning, Jiang He, Weiqing Wang, Yufang Bi, Dalong Zhu, Jiguang Wang, Shengdi Chen, Yu Xu, BPROAD coordinating team at Ruijin Hospital, Lawrence J. Appel, William C. Cushman, Vivian A. Fonseca, Jeff D. Williamson, David M. Reboussin, Yaling Han, Hongbing Shen, Minghui Zhao, Hui Wang

**Affiliations:** ^1^ Ruijin Hospital Shanghai; ^2^ Tulane University New Orleans; ^3^ Nanjing Drum Tower Hospital Nanjing; ^4^ Johns Hopkins University Baltimore USA; ^5^ University of Tennessee Memphis USA; ^6^ Tulane University New Orleans USA; ^7^ Wake Forest University Winston‐Salem USA; ^8^ General Hospital of Northern Theater Command Shenyang China; ^9^ Nanjing Medical University Nanjing China; ^10^ Peking University First Hospital Beijing China; ^11^ Shanghai Jiaotong University Shanghai China

**Keywords:** blood pressure, cardiovascular disease, randomized controlled trial, type 2 diabetes, 血压, 心血管疾病, 随机对照试验, 2型糖尿病

## Abstract

**Background:**

Diabetes and hypertension are major modifiable risk factors for cardiovascular disease. Previous clinical trials have demonstrated that intensive blood pressure reduction lowers the risk of cardiovascular disease and all‐cause mortality compared to standard blood pressure reduction among patients without diabetes. However, optimal levels of blood pressure control in patients with diabetes remain uncertain.

**Methods:**

The Blood Pressure Control Target in Diabetes (BPROAD) study is a multicenter, randomized controlled trial conducted in mainland China. We plan to enroll 12 702 participants aged ≥50 years with type 2 diabetes, an increased cardiovascular risk, and systolic blood pressure ≥130 mm Hg from 150 study centers. Participants are randomly assigned to intensive (a systolic target of <120 mm Hg) or standard (a systolic target of <140 mm Hg) blood pressure treatment group. Participants will be followed monthly for blood pressure management in the first 3 months and then every 3 months afterward. The primary study outcome is a composite of major cardiovascular events including nonfatal myocardial infarction, nonfatal stroke, treated or hospitalized heart failure, and cardiovascular death. Data will be collected every 3 months for up to 5 years and a blinded outcome committee will adjudicate all clinical outcomes. The BPROAD study is designed to have 90% statistical power to detect a 20% reduction in the primary study outcome at a two‐sided significance level of 0.05.

**Conclusions:**

The BPROAD study will provide important evidence as to whether intensive blood pressure management has additional benefits on cardiovascular disease and all‐cause mortality among patients with type 2 diabetes.

## Graphical abstract

Highlights

- The Blood Pressure Control Target in Diabetes (BPROAD) study is a multicenter, randomized controlled trial conducted in mainland China to test the hypothesis of whether intensive blood pressure (BP) management (systolic BP <120 mm Hg) has additional benefits over standard BP management (systolic BP <140 mm Hg) over a follow‐up period of up to 5 years in patients with type 2 diabetes.
- Findings from the BPROAD study will guide the development of clinical guidelines for blood pressure management aimed at reducing cardiovascular disease burden.

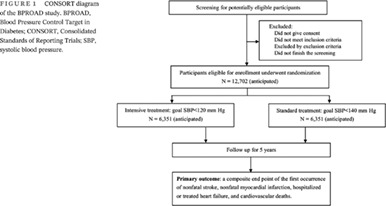

## 摘要

背景:糖尿病和高血压是心血管疾病两个主要可改变的风险因素。既往临床试验证明，与标准血压降低相比，强化降压可以减少心血管疾病和全因死亡风险，特别是在没有糖尿病的患者中。然而，仍然未确定糖尿病患者血压控制的最佳水平。

方法:BPROAD研究是一项在中国大陆进行的多中心随机对照试验。计划从150个研究中心招募12,702名年龄≥50岁、有心血管风险、收缩压≥130mmHg的2型糖尿病患者。参与者被随机分配到强化组(收缩压目标<120mmHg)或标准组(收缩压目标<140mmHg)。参与者在前三个月每月接受一次血压管理，之后每三个月一次。主要研究结果是一个复合主要心血管事件，包括非致命性心肌梗死、非致命性中风、治疗或住院的心力衰竭和心血管死亡。数据每三个月收集一次，最多五年，一个盲态的结果委员会对所有临床结果进行裁定。BPROAD研究旨在双侧显著性水平为0.05的情况下，具有90%的统计效力来检测主要研究结果减少20%。

结论:BPROAD研究将为2型糖尿病患者提供重要证据，以确定强化血压管理是否对心血管疾病和全因死亡有额外的好处。

## 1 BACKGROUND AND RATIONALE

The number of adults with diabetes in the world has nearly quadrupled from 1980 to 2014, corresponding to a growing and aging population.^1^ The International Diabetes Federation has estimated a total of 463 million (9.3%) adults aged 20–79 years worldwide currently living with diabetes, and the total number is predicted to rise to 578 million (10.2%) by 2030 and to 700 million (10.9%) by 2045.^2^ Meanwhile, the prevalence of diabetes in mainland China increased from 0.67% in 1980 to 12.8% in 2017, with an estimated population with diabetes at 129.8 million.^3^

Hypertension is a common comorbidity in patients with type 2 diabetes. Patients with a history of diabetes have a higher prevalence (66.3% vs 21.9%) and a lower control rate (4.7% vs 19.6%) of hypertension than those with normal glucose regulation in Chinese adults.^4^, ^5^ Findings from prospective cohort studies suggest that 35%–75% of the cardiovascular risk in patients with diabetes can be attributed to hypertension.^6^

Meta‐analyses of previous randomized clinical trials have demonstrated the benefit of lowering blood pressure (BP) to <140/<90 mm Hg in patients with diabetes.^7^, ^8^ The Action to Control Cardiovascular Risk in Diabetes (ACCORD) trial is the only randomized trial that compared a systolic BP lower than 140 mm Hg with a lower systolic BP goal in people with diabetes. No significant difference in cardiovascular events (hazard ratio [HR] 0.88; 95% confidence interval [CI] 0.73–1.06) or all‐cause mortality (HR 1.07, 95% CI 0.85–1.35) was observed between intensive treatment (systolic BP goal <120 mm Hg) and standard treatment (systolic BP goal <140 mm Hg) among 4733 participants with type 2 diabetes. The insufficient occurrence of primary outcome in the control group (2.09% per year observed vs 4.0% per year anticipated) and interaction between glucose and blood pressure control (*p* for interaction = .08) were thought to contribute to the insignificant finding.^9^ It is noteworthy that intensive BP lowering treatment significantly reduced the risk of cardiovascular events in the subgroup of standard glycemic control in ACCORD. Furthermore, the Systolic Blood Pressure Intervention Trial (SPRINT) showed that compared to standard treatment (systolic BP goal <140 mm Hg), intensive treatment (systolic BP goal <120 mm Hg) significantly lowered major adverse cardiovascular events (HR 0.75, 95% CI 0.64–0.89) and all‐cause mortality (HR 0.73, 95% CI 0.60–0.90) among 9361 patients with a systolic BP ≥130 mm Hg and an increased cardiovascular risk.^10^ However, patients with diabetes were excluded from this trial. Meta‐analyses of randomized trials of antihypertensive treatment in patients with diabetes revealed that BP lowering was associated with improved cardiovascular morbidity and mortality only among those with baseline systolic BP ≥140 mm Hg.^11^, ^12^

Therefore, the optimal systolic BP treatment goal for the prevention of cardiovascular disease (CVD) and all‐cause mortality among patients with diabetes remains unknown. The Blood Pressure Control Target in Diabetes (BPROAD) study was designed to test the hypothesis of whether intensive BP management to achieve a systolic BP target of <120 mm Hg has additional benefits over standard BP management to achieve a systolic BP target of <140 mm Hg over a period of up to 5 years in patients with type 2 diabetes. The findings from this study will help in the development of clinical guidelines for BP management among patients with diabetes.

## 2 STUDY DESIGN

**FIGURE 1:**
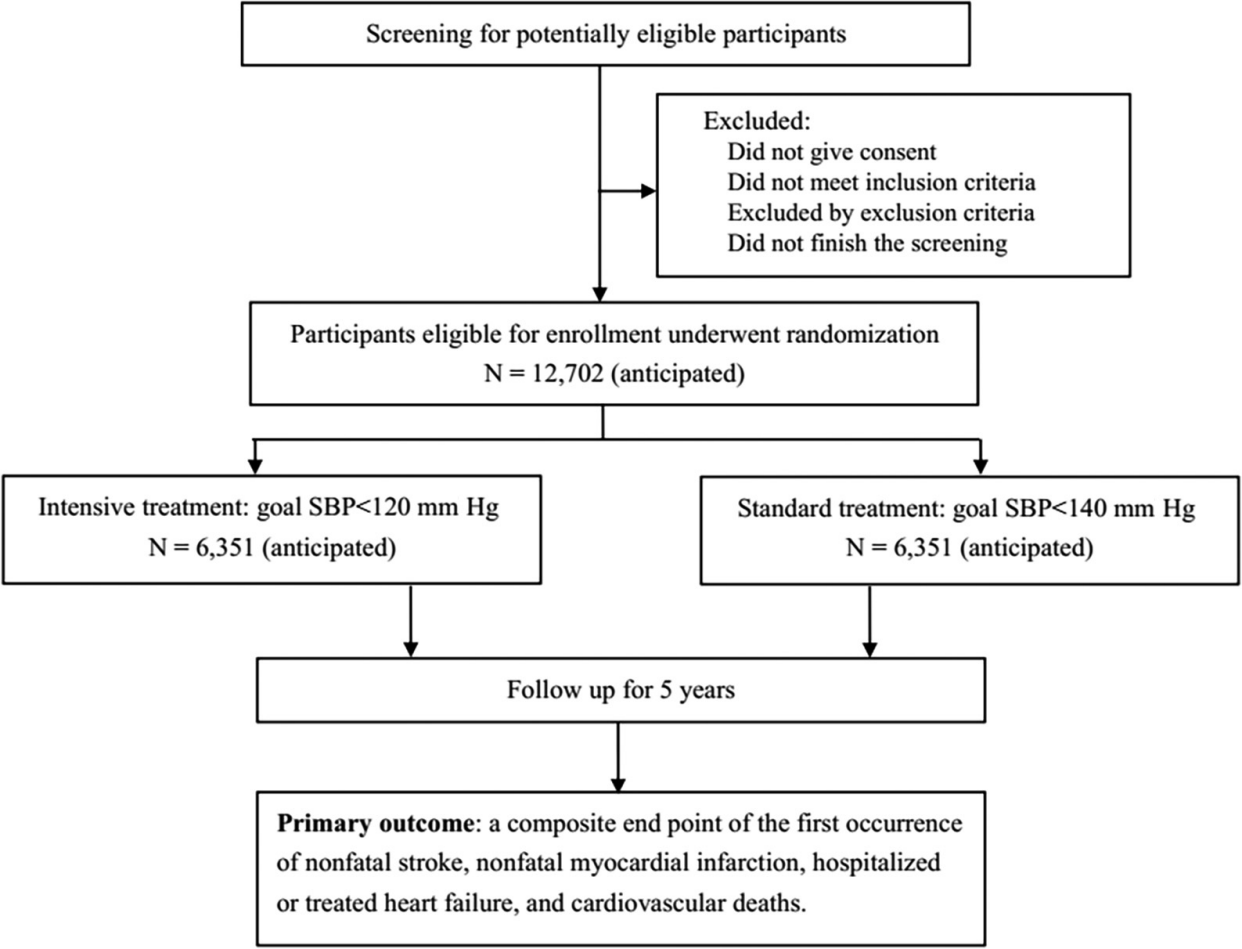
CONSORT diagram of the BPROAD study. BPROAD, Blood Pressure Control Target in Diabetes; CONSORT, Consolidated Standards of Reporting Trials; SBP, systolic blood pressure.

The BPROAD study is a multicenter, outcome assessor blinded, randomized controlled trial (ClinicalTrials.gov Identifier: NCT03808311) that will enroll 12 702 type 2 diabetes patients with elevated systolic BP and increased CVD risk from 150 study centers across mainland China to undergo randomly antihypertensive treatment achieving systolic BP <120 mm Hg or systolic BP <140 mm Hg for up to 5 years (Figure 1). The primary outcome is a composite of major CVD events including nonfatal myocardial infarction, nonfatal stroke, treated or hospitalized heart failure, and cardiovascular death.

### 2.1 Study setting and participants

Participating centers are recruited from all over mainland China. To be eligible, a BPROAD participating center should (a) be a government‐approved secondary or tertiary hospital, (b) have ≥500 type 2 diabetes patients per year, (c) have a well‐accredited medical laboratory for clinical measurements, (d) have at least two study physicians with expertise in cardiometabolic diseases and one study assistant, and (e) be willing to participate in the trial and comply with trial protocol.

Participants are recruited at each participating center. The inclusion and exclusion criteria of BPROAD participants are presented in Table 1. The trial will be conducted among type 2 diabetes patients aged ≥50 years with elevated systolic BP and increased risk of CVD. Elevated systolic BP was defined as 130–180 mm Hg with antihypertensive medications or ≥140 mm Hg without medications. There are no diastolic BP inclusion criteria. A participant who presents without BP medications should have documentation of systolic BP ≥140 mm Hg on two visits within 3 months prior to the randomization visit in order to be eligible for the trial.

**TABLE 1:** Inclusion and exclusion criteria of study participants.

Inclusion criteria
Age ≥50 years
2 Type 2 diabetes
3 Systolic blood pressure
≥140 mm Hg without taking any antihypertensive drug; or 130–180 mm Hg on 1 antihypertensive drug; or 130–170 mm Hg on up to 2 antihypertensive drugs; or 130–160 mm Hg on up to 3 antihypertensive drugs; or 130–150 mm Hg on up to 4 antihypertensive drugs
4 Increased risk of CVD (one or more of the following):
Previous history of clinical CVD (≥3 months) Stroke Myocardial infarction PCI or CABG Carotid endarterectomy or carotid stenting Peripheral artery disease with revascularization Acute coronary syndrome
Subclinical CVD within 3 years Microalbuminuria ≥50% stenosis of a coronary, carotid, or lower extremity artery Coronary artery calcium score ≥ 400 Agatston units Ankle brachial index ≤0.90 Left ventricular hypertrophy If microalbuminuria is the only criterion satisfied for increased risk of cardiovascular disease, replication to confirm is required.
Two or more CVD risk factors Current cigarette smoking BMI ≥28 kg/m^2^ or waist circumference ≥90 cm (in men) or ≥ 85 cm (in women) Current lipid‐lowering medications or LDL‐C ≥130 mg/dL (3.38 mmoL/L) Current lipid‐lowering medications or HDL‐C <40 mg/dL (1.04 mmoL/L) Current lipid‐lowering medications or triglycerides ≥150 mg/dL (1.69 mmoL/L) eGFR: 30–59 mL/min/1.73 m^2^
B. Exclusion criteria
1 History consistent with type 1 diabetes 2 Known secondary cause of hypertension (including procedures for renal artery disease) 3 One‐minute standing systolic BP <110 mm Hg 4 Arm circumference too large to allow accurate BP measurement with available devices 5 Cardiovascular event or procedure (as defined above as clinical CVD for study entry) or hospitalization for unstable angina within the past 3 months 6 Symptomatic heart failure or left ventricular ejection fraction (by any method) <35% within the past 6 months 7 ALT or AST levels more than twice the upper limit of the normal range or active liver diseases 8 Dialysis or renal transplantation, or eGFR <30 mL/min/1.73 m^2^ or serum creatinine >2.0 mg/dL 9 Proteinuria 24‐h urinary protein excretion ≥1 g/day, or 24‐h urinary albumin excretion ≥600 mg/day, or Spot urine protein/creatinine ratio ≥1 g/g, or Albumin/creatinine ratio ≥600 mg/g 10 Previous diagnosis of polycystic kidney disease or glomerulonephritis 11 A medical condition likely to limit survival to less than 5 years 12 Any factors judged by the clinical team to be likely to limit adherence to interventions 13 Failure to obtain informed consent from participant 14 Currently participating in another intervention study 15 Currently living with another BPROAD participant 16 Pregnancy, currently trying to become pregnant, or of child‐bearing potential and not using birth control

Abbreviations: ALT, alanine aminotransferase; AST, aspartate aminotransferase; BMI, body mass index; BPROAD, Blood Pressure Control Target in Diabetes; CABG, coronary artery bypass graft; CVD, cardiovascular disease; ECG, electrocardiogram; eGFR, estimated glomerular filtration rate; HDL‐C, high‐density lipoprotein cholesterol; LDL‐C, low‐density lipoprotein cholesterol; PCI, percutaneous coronary intervention.

All study documents such as the study protocol, manual of procedures, and informed consent were approved in advance by the Institutional Review Board (IRB) of Ruijin Hospital, as well as by the IRBs of participating study centers. Written informed consent is obtained from all study participants.

### 2.2 Intervention

**FIGURE 2:**
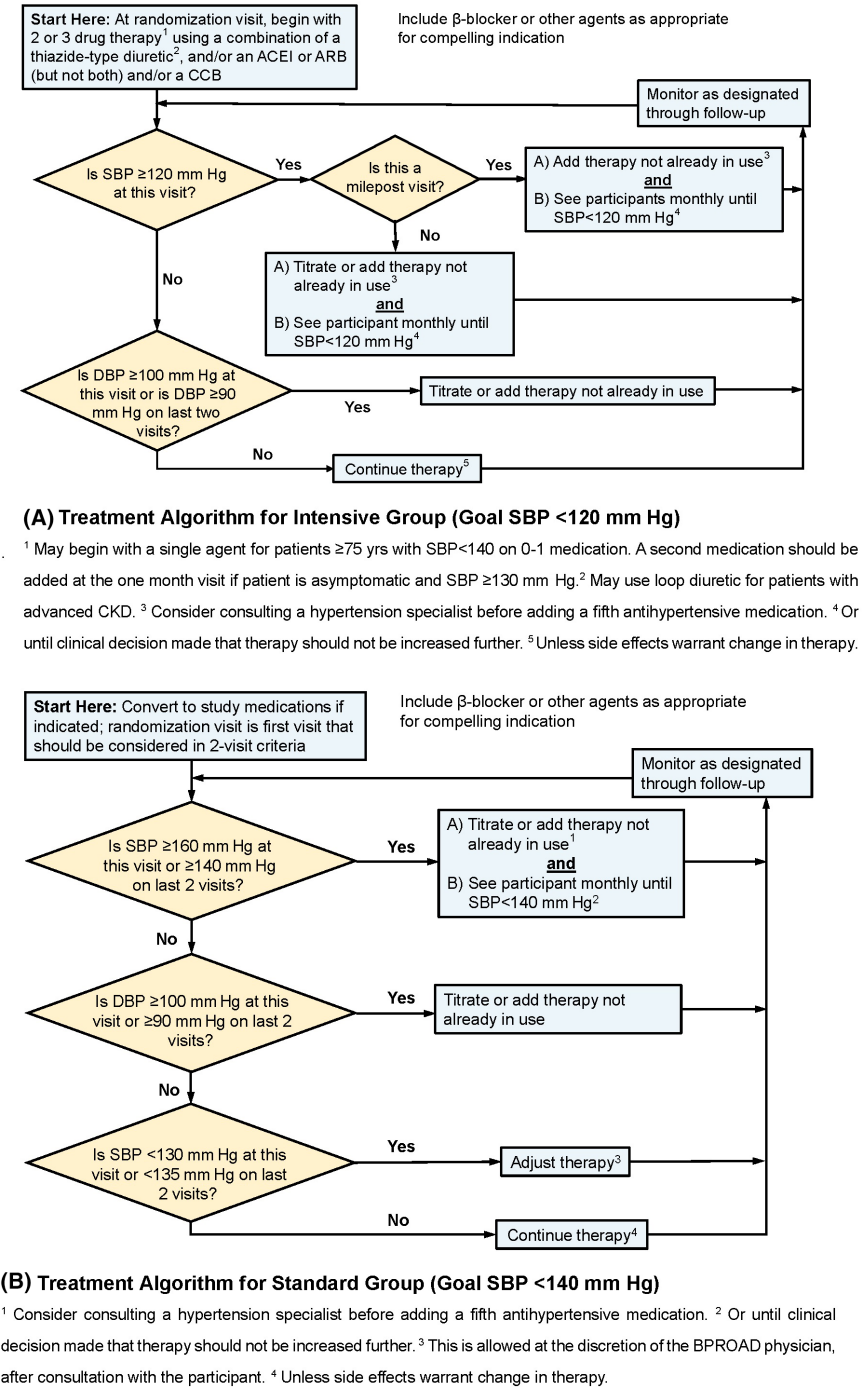
Blood pressure treatment algorithms for the intensive and standard treatment groups. ACEI, angiotensin‐converting enzyme‐inhibitor; ARB, angiotensin receptor blocker; CCB, calcium channel blocker; CKD, chronic kidney disease; DBP, diastolic blood pressure; SBP, systolic blood pressure.

Participants eligible for the BPROAD study will be randomized to one of the two systolic BP goals: <120 mm Hg for the intensive treatment group and <140 mm Hg for the standard treatment group (Figure 2). The targeted mean systolic BP difference between the two randomized groups will be ≥15 mm Hg. One or more medications from the following classes of antihypertensive agents will be intended for use to achieve intervention goals in randomization groups: angiotensin‐converting enzyme‐inhibitors (ACEI), angiotensin receptor blockers (ARB), calcium channel blockers (CCB), thiazide‐type diuretics, loop diuretics, potassium‐sparing diuretics, beta‐blockers, alpha1‐receptor blockers, sympatholytics, and direct vasodilators. The BP treatment protocol is flexible in terms of the choice and doses of antihypertensive medications. The new 2018 Chinese Guidelines for the Prevention and Management of Hypertension, along with relevant new scientific developments, will be recommended to guide drug choices.^13^

For both randomized groups, protocol visit frequency will be monthly for the first 3 months after randomization, then every 3 months thereafter. Monthly visits will continue in the intensive treatment group until systolic BP <120 mm Hg (or no more titration planned) and in the standard treatment group until systolic BP <140 mm Hg (or no more titration planned). A PRN (Latin *pro re nata*, “as needed”) visit will be scheduled if participants report adverse events, or for monitoring significant medication changes and safety alerts, or other clinical issues.

For most participants in the intensive treatment group, a two‐ or three‐drug regimen of a diuretic and either an ACEI or ARB and/or a CCB should be initiated at randomization. Drug doses should be increased and/or extra antihypertensive agents should be added at monthly visit until the participant's goal of <120 mm Hg has been reached or the study physician decides no further antihypertensive agents may be added. Milepost visits based on the ACCORD and SPRINT experience for the intensive treatment group will be scheduled every 6 months beginning at the 6‐month visit throughout the follow‐up, to assist in reaching systolic BP <120 mm Hg. If the systolic BP ≥120 mm Hg at a milepost visit, an additional agent from an antihypertensive drug class different from what is being taken should be added.

A systolic BP of 135–139 mm Hg should be achieved in as many participants as possible in the standard treatment group to generate the planned differences in systolic BP between treatment groups. Changes in doses and/or choices of anti‐hypertensive agents are indicated if systolic BP is ≥160 mm Hg at a single visit or is ≥140 mm Hg at two consecutive visits. Down‐titration will be recommended after full discussion with the patient if the systolic BP is <130 mm Hg at a single visit or <135 mm Hg at two consecutive visits.

Once the systolic BP goal is achieved, the antihypertensive regimen should be intensified if diastolic BP remains ≥100 mm Hg at a single visit or ≥90 mm Hg at two consecutive visits to achieve diastolic BP <90 mm Hg.

The background therapy recommendations such as glucose and lipid management are based on the clinical guidelines of the Chinese Diabetes Society and the American Diabetes Association.^14^, ^15^ The delivery of these background therapies will be left up to the participants' own clinicians. Participants undergo BP treatment intervention during study visits while attending clinics for concomitant treatment including glucose and lipid management as their usual care to minimize the differences in the effects of nonstudy strategies on CVD outcomes between intervention groups.

### 2.3 Outcomes

Occurrence of study outcomes will be collected every 3 months beginning at the 3‐month visit and throughout the follow‐up. The physician who collects outcome data is masked to treatment assignment. Photocopies of inpatient record, discharge summary, electrocardiogram, and imaging reports, etc. will be obtained for outcome adjudication by members of an outcome assessment committee who are unaware of treatment assignment. At least two committee members will review the medical records independently. Clinical events occurring during follow‐up will be ascertained equally in both treatment groups.

BPROAD study outcomes are listed in Table 2. The primary outcome for the BPROAD study is the composite end point of the first occurrence of nonfatal stroke, nonfatal myocardial infarction, hospitalized or treated heart failure, and cardiovascular death. The following subgroups are prespecified to test whether the effect of an intervention achieving systolic BP <120 mm Hg versus systolic BP <140 mm Hg on the primary study outcome is consistent in participants with different characteristics.

- Age at baseline (<65 vs ≥65 years; <80 vs ≥80 years).
- Men vs women.
- Systolic BP levels at baseline (tertiles).
- Previous CVD.
- Previous chronic kidney disease (CKD, estimated glomerular filtration rate [eGFR] <60 mL/min/1.73 m^2^).
- Glycated hemoglobin (HbA1c) at baseline (tertiles).
- Diabetes duration at baseline (< vs ≥ the median).
- High BP duration at baseline (< vs ≥ the median).

**TABLE 2:** Study outcomes.

Primary outcome
Major adverse cardiovascular events defined as the composite endpoint of the first occurrence of nonfatal stroke, nonfatal MI, hospitalized or treated heart failure, and cardiovascular death.
B. Secondary outcomes
A composite of the primary outcome and all‐cause mortality Macrovascular outcome including the composite primary outcome, hospitalized unstable angina, and all cardiovascular revascularization procedures Major coronary artery diseases including nonfatal MI, hospitalized unstable angina, revascularization of coronary arteries, and deaths due to coronary artery diseases Total MI including fatal and nonfatal MI Total stroke including fatal and nonfatal stroke Ischemic stroke Hemorrhagic stroke Hospitalized or treated heart failure or heart failure death Cardiovascular death Total mortality Cognitive function Health related quality of life C. Kidney outcomes Progression of CKD defined as the composite of a 50% decrease in eGFR, development of end‐stage renal disease requiring chronic dialysis or kidney transplantation, or eGFR <15 mL/min/1.73 m^2^ in patients with CKD at baseline. Development of CKD defined as a >30% decrease in eGFR and eGFR <60 mL/min/1.73 m^2^ among patients without CKD at baseline. Incident albuminuria defined as a doubling of urinary ACR from a value of <10 mg/g to a value of >10 mg/g in all patients with or without CKD. D. Other outcomes All cardiovascular revascularization procedures Hospitalized unstable angina Retinopathy Transient ischemic attack Left ventricular hypertrophy diagnosed by ECG Atrial fibrillation or flutter All cancers Cost‐effectiveness measures

Abbreviations: ACR, albumin‐to‐creatinine ratio; BP, blood pressure; CKD, chronic kidney disease; ECG, electrocardiogram; eGFR, estimated glomerular filtration rate; MI, myocardial infarction.

Secondary outcomes, cognitive outcomes, kidney outcomes, and other outcomes are specified in Table 2.

### 2.4 Safety monitoring

Patient safety will be carefully monitored. Safety outcomes include serious adverse events (SAEs) and selected AEs. SAEs are adverse events that are fatal or life threatening, result in significant or persistent disability, require or prolong hospitalization, or are important medical events that investigators judge to represent significant hazards or harm to research participants. In addition, a selected list of other important events that lead to emergency room visits will also be reported in BPROAD. The selected AEs include symptomatic hypotension, arrhythmia, acute kidney dysfunction, electrolyte abnormalities, injurious falls, syncope, and unexpected events for which the investigator believes that the BPROAD intervention might cause the event or might contribute to the immediate cause of the event. Participants will be queried for SAEs and selected AEs at each follow‐up visit. Participants are also encouraged to report SAEs and selected AEs between visits.

In addition, an independent Data and Safety Monitoring Board (DSMB) will be responsible for monitoring signs of adverse trends in morbidity/mortality and treatment‐related SAEs. The BPROAD DSMB members are experts in antihypertensive clinical trials, diabetes, cardiology, nephrology, neurology, and biostatistics. Before each DSMB meeting, the Coordinating Center at Ruijin Hospital will prepare and provide data on SAEs and selected AEs and any other safety information requested by the DSMB for discussion during open and closed sessions. The DSMB members vote during the closed sessions on recommendations to continue or terminate the study based on safety or efficacy data.

### 2.5 Data collection

Outcome data collection starts at 3 months after random assignment and every 3 months thereafter in both treatment groups. Participants in both treatment groups will be queried on the occurrence of study outcomes on the same schedule, which is every 3 months.

Assessments performed at the various visits include questionnaires, physical examinations, blood and urine collection, lab measurements, etc. (Table 3). The same schedule will be used for both randomization groups. Baseline characteristics include socio‐demographics, medical history, concomitant medications, anthropometrics, BP levels, fasting plasma glucose, HbA1c, laboratory chemistry, cognitive function, and health‐related quality of life measurements. BP will be measured based on the recommendations by the American Heart Association.^16^ Participants are advised to avoid cigarettes, alcohol, coffee/tea, and exercise for ≥30 min before BP measurements. BP is measured three times after 5 min of seated rest with 1‐min intervals between measurements, at the presence of observer. The automated device (Omron HEM‐907) will be used and one of three cuff sizes (pediatric, regular adult, or large) will be chosen on the basis of each participant's arm circumference. The mean of the three measurements will be used.

**TABLE 3:** Measurement and data collection schedule.

	Bs/Rz	Month 3	Month 6	Month 9	Year 1 AV	Year 1 QV	Year 2 AV	Year 2 QV	Year 3 AV	Year 3 QV	Year 4 AV	Year 4 QV	Year 5 AV or close‐out
Questionnaires													
Medical history, SES variables	X												
Lifestyle factors	X						X				X		
Adherence and adverse events		X	X	X	X	X	X	X	X	X	X	X	X
Study outcomes		X	X	X	X	X	X	X	X	X	X	X	X
Physical examinations													
Seated BP, pulse and medication adjustment	X	X	X	X	X	X	X	X	X	X	X	X	X
Standing BP	X		X		X		X		X		X		X
Weight	X	X	X	X	X	X	X	X	X	X	X	X	X
Height	X												
Waist and hip circumference	X				X		X		X		X		X
ECG	X				X		X		X		X		X
Lab measurements													
Liver and kidney function, electrolytes, glucose^a^	X	X	X		X		X		X		X		X
Blood routine, 24‐h urinary electrolytes, protein, and creatinine excretion^a^	X				X		X		X		X		X
Serum creatinine, lipid profile, and HbA1c^b^	X				X		X		X		X		X
Urinary albumin and creatinine^b^	X				X		X		X		X		X
Cognitive function	X				X		X		X		X		X
Health related quality of life	X				X		X		X		X		X

Abbreviations: AV, annual visits; BP, blood pressure; Bs/Rz, baseline and randomization visits; ECG, electrocardiogram; QV, quarterly visits; SES, socioeconomic status.

^a^ Measured at local sites.

^b^ Measured at the central lab of the Coordinating Center.

### 2.6 Randomization and blinding

The randomization will be conducted on stratification of study centers. At each study center, block randomization will be used with randomly selected block sizes of two, four, and six. Study investigators at each study center will be informed of the assignment of specific participants via the electronic data capture system.

Because BPROAD is a trial comparing two different levels of systolic BP control in patients with diabetes, blindness is not possible for study participants and study physicians. However, study staff collecting information on study outcomes will be masked to treatment assignment. In addition, the study outcome committee as well as statisticians will also be masked to treatment assignment.

### 2.7 Sample size and statistical analysis

#### 2.7.1 Sample size

The BPROAD study has a single primary outcome (composite major CVDs) and several key secondary outcomes. The sample size calculation is based on the primary outcome and use of the following assumptions:

- Event rate of composite major CVDs of 2.0% per year among patients in the standard treatment group
- 20% effect size for the intervention (HR of 0.80)
- Two‐year uniform recruitment period and total study length of 5 years (defined by the time between randomization of the first participant and study closeout)
- 2% per year loss to follow‐up rate
- Two‐sided significance test at the 5% level, and a statistical power of 90%

Based on these assumptions, the BPROAD study will need to enroll a total of 12 702 patients with equal numbers in both treatment groups.

#### 2.7.2 Statistical analysis

The primary analysis will be based on participants' randomization assignment regardless of their achieved BP levels (intention‐to‐treat analysis). Follow‐up time will be calculated from the date of randomization to the date of the event ascertainment for patients with event occurrence, the date of the trial completion for patients without event occurrence, the date of death for patients who died during follow‐up, or the date of last contact for patients lost to follow‐up. Cox proportional hazards regression will be used to compare the time from randomization to the first CVD event between the randomization groups with two‐sided tests at the 5% level of significance. The model will include an indicator for intervention as its sole predictor variable. Study center at randomization will be a stratifying factor.

Tests of secondary outcomes will be conducted with models similar to those used in the primary analysis. These will be reported with 95% CIs and nominal *p* values without an adjustment for multiple comparisons. Interactions between treatment effect and prespecified subgroups will be assessed with a likelihood‐ratio test using Hommel adjusted *p* values. The multiple imputation techniques will be used in the sensitivity analysis of the primary and secondary outcomes.

## 3 QUALITY CONTROL

The BPROAD Manual of Procedures (MOP) includes all the details of trial procedures. We use the BPROAD MOP to train study investigators and staff and as a reference during trial preparation and conduct. Key study procedures are standardized, such as standard forms, equipment, and procedures in the study centers for BP measurement, the use of a central laboratory, ECG reading center, and outcome assessment committee at the Coordinating Center.

Training of staff is important to standardize procedures and assure data quality. We organize up to 10 training sessions at different regions across mainland China. Staff members from study centers within the regions are convened in a single, centrally administered face‐to‐face training session. We conduct a second face‐to‐face training at individual study center before screening of the first participant in that study center. Training videos are available for study staff to watch at any time. In addition, the Coordinating Center organizes yearly refresher training sessions to all study centers.

Data are collected using an electronic data capture system. The Coordinating Center is responsible for data monitoring and query reports. Study center staff is responsible for reviewing and resolving the data queries in a timely manner. Routine (eg, monthly) quality control reports will be developed by the Coordinating Center and will be distributed to all study centers on measures of process, impact, and outcomes.

Study samples are collected and centrifuged, aliquoted, and stored at −80 degrees before shipment in dry ice to the central laboratory within 3 months after collection. The central laboratory participates in the American College of Pathologists' Laboratory Accreditation Program and is reviewed and accredited on a regular basis. Samples are measured according to standard procedures and quality control requirements to ensure high‐quality measurement data. Samples that do not undergo measurement are stored at −80 degrees in the Coordinating Center for future research.

## 4 BPROAD DURING COVID‐19

The first cases of COVID‐19 infection were reported in China in late December 2019. Wuhan, the city most heavily infected, was locked down during 23 January–8 April 2020. This was regarded as the time period when the COVID‐19 epidemic was severe in mainland China. Radical public health measures including quarantine and social distancing were strictly implemented; therefore, in‐person visits of BPROAD participants with their doctors/investigators became unlikely.

On 10 February 2020, the BPROAD Coordinating Center developed an urgent response plan to the outbreak of COVID‐19 according to the China Forums of Clinical Research Capacity Building and Human Research Participants Protection Consensus on Clinical Trial Management under First‐level Response to Major Public Health Emergencies (Infectious Diseases).^17^ The response plan recommended to (a) cancel or suspend all screening, baseline/randomization, and the first yearly visits; (b) collect trial data by telephone interviews at the scheduled date; and (c) complete data collection within 2 weeks of the scheduled date for all BPROAD study centers. BP levels were obtained using home BP monitoring (HBPM). Training and instructions on HBPM were provided to each participants for them to measure and record BP levels at home accurately and report to their doctors/investigators. Medications were adjusted at the discretion of the study physician taking into account the target systolic BP and the validity of BP numbers provided by the participant. The same targets for home BP and clinic BP were recommended. A drug prescription for 90 days on each fill is encouraged by the central government for chronic diseases including hypertension during the outbreak. Although BPROAD in‐person visit is not required, patients can still go to clinics for prescriptions if drug refill is needed. Information on study outcomes and SAEs was asked and supporting clinical materials are obtained afterward whenever possible. The response plan was approved by the IRB of Ruijin Hospital and the DSMB, and was implemented until 31 March 2020.

## 5 STUDY ENROLLMENT

**FIGURE 3:**
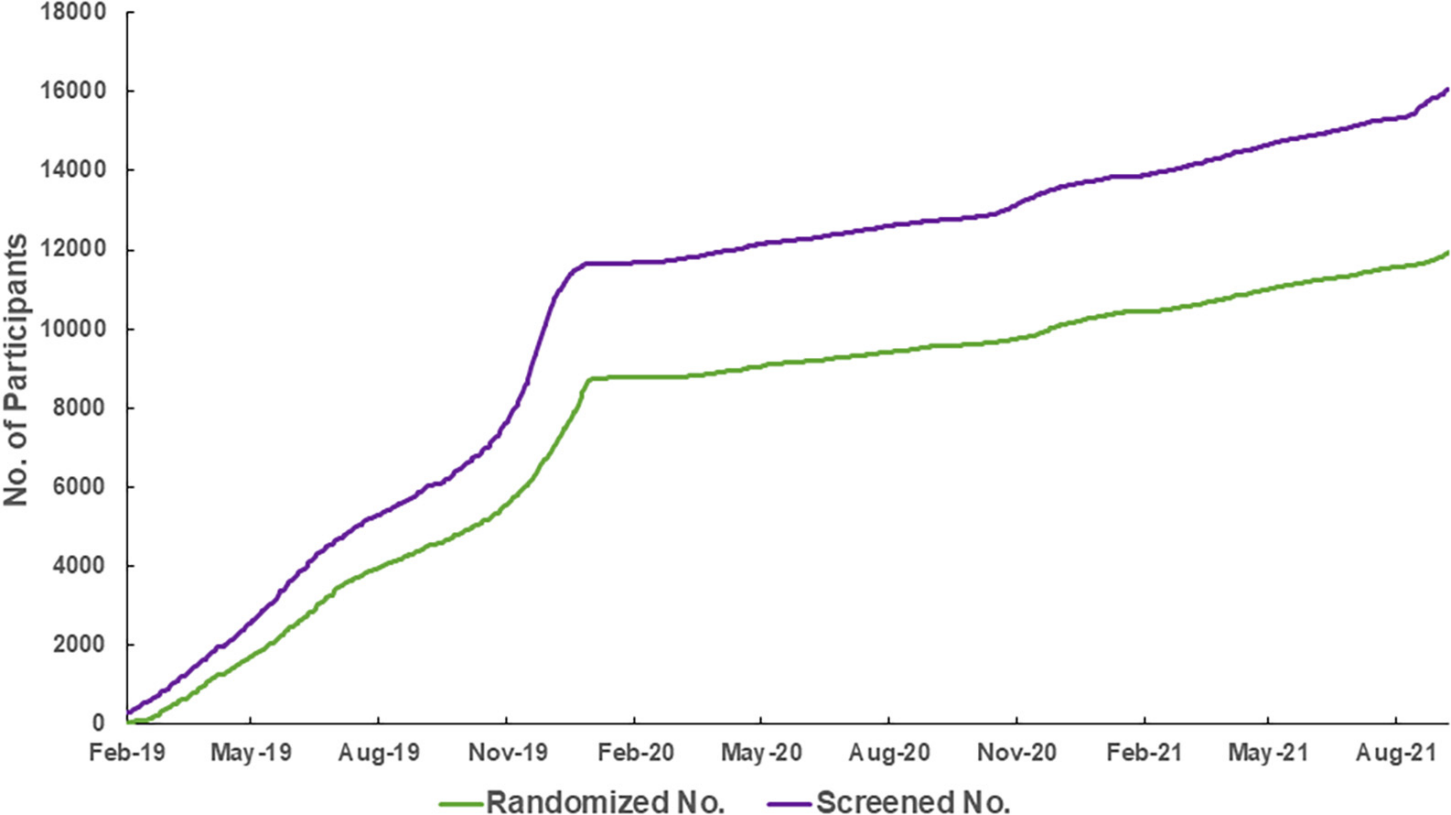
Study enrollment till 30 September 2021.

BPROAD participants are recruited from diabetes clinics across mainland China. The first BPROAD participant was randomized on 24 February 2019. Participant recruitment was ahead of schedule until the outbreak of COVID‐19 in mainland China in January 2020 (Figure 3), after which participant recruitment was slowed down substantially. By 30 September 2021, a total of 16 047 patients have been screened and 11 927 participants have been enrolled, although the planned completion of recruitment was on 24 February 2021. We anticipate to complete recruitment by the end of 2021.

## 6 CONCLUSIONS

Previous studies have demonstrated the benefits of BP reduction to <140 mm Hg on lowering the risk of CVD and all‐cause mortality among subjects with or without diabetes.^7^, ^8^ The SPRINT trial revealed additional benefits of further BP reduction to <120 mm Hg among subjects without diabetes.^10^ However, the ACCORD trial failed to support further BP reduction to <120 mm Hg among subjects with diabetes. The less‐than‐anticipated occurrence of major CVD events in the control group might play a part in the null finding.^9^ The appropriate target for systolic BP reduction in patients with diabetes remains uncertain.

The BPROAD study is designed to answer the question whether there are additional cardiovascular benefits to lower systolic BP to <120 mm Hg compared with systolic BP to <140 mm Hg among patients with diabetes over a follow‐up period of up to 5 years with an estimated 90% statistical power. Together with the SPRINT and ACCORD trials (Table 4), as well as other ongoing BP target trials worldwide, the BPROAD study will have essential impacts on public health and clinical practice of BP management.

**TABLE 4:** ACCORD BP, SPRINT, and BPROAD trials.

	ACCORD BP	SPRINT	BPROAD
Study period	2003–2009	2010–2015	2019~
Study design	Factorial assignment (2‐by‐2)	Parallel assignment	Parallel assignment
Masking	Open label (blinded outcome adjudication committee)	Open label (blinded outcome adjudication committee)	Open label (blinded outcome adjudication committee)
Participants			
Number	4733	9361	12 702 (anticipated)
Country	United States and Canada	United States	China
Age	40 to 79 years	≥50 years	≥50 years
Eligibility criteria	Type 2 diabetes, elevated systolic BP, and increased CVD risk	Elevated systolic BP, and increased CVD risk excluding those with diabetes or previous stroke	Type 2 diabetes, elevated systolic BP, and increased CVD risk
Intervention	Systolic BP <120 mm Hg vs systolic BP <140 mm Hg	Systolic BP <120 mm Hg vs systolic BP <140 mm Hg	Systolic BP <120 mm Hg vs systolic BP <140 mm Hg
Mean intervention duration	4.7 years	3.26 years (median)	4 years (anticipated)
Primary outcome	A composite of nonfatal myocardial infarction, nonfatal stroke, and CVD death	A composite of myocardial infarction, acute coronary syndrome, stroke, acute decompensated heart failure, and CVD death	A composite of non‐fatal stroke, non‐fatal myocardial infarction, hospitalized or treated heart failure, and CVD death

Abbreviations: ACCORD, Action to Control Cardiovascular Risk in Diabetes; BP, blood pressure; BPROAD, Blood Pressure Control Target in Diabetes; CVD, cardiovascular disease; SPRINT, Systolic Blood Pressure Intervention Trial.

## DISCLOSURE

None.
